# Warning Signs of Potential Black Swan Outbreaks in Infectious Disease

**DOI:** 10.3389/fmicb.2022.845572

**Published:** 2022-02-24

**Authors:** Nileena Velappan, Katie Davis-Anderson, Alina Deshpande

**Affiliations:** Biosecurity and Public Health, Bioscience Division, Los Alamos National Laboratory, Los Alamos, NM, United States

**Keywords:** black swan event, infectious disease, outbreak analysis, prediction and forecasting, visual analytic

## Abstract

Black swan events in infectious disease describe rare but devastatingly large outbreaks. While experts are skeptical that such events are predictable, it might be possible to identify the warning signs of a black swan event. Specifically, *following* the initiation of an outbreak, key differentiating features could serve as alerts. Such features could be derived from meta-analyses of large outbreaks for multiple infectious diseases. We hypothesized there may be common features among the pathogen, environment, and host epidemiological triad that characterize an infectious disease black swan event. Using Los Alamos National Laboratory’s tool, Analytics for Investigation of Disease Outbreaks, we investigated historical disease outbreak information and anomalous events for several infectious diseases. By studying 32 different infectious diseases and global outbreaks, we observed that in the past 20–30 years, there have been potential black swan events in the majority of infectious diseases analyzed. Importantly, these potential black swan events cannot be attributed to the first introduction of the disease to a susceptible host population. This paper describes our observations and perspectives and illustrates the value of broad analysis of data across the infectious disease realm, providing insights that may not be possible when we focus on singular infectious agents or diseases. Data analytics could be developed to warn health authorities at the beginning of an outbreak of an impending black swan event. Such tools could complement traditional epidemiological modeling to help forecast future large outbreaks and facilitate timely warning and effective, targeted resource allocation for mitigation efforts.

## Introduction

Disease outbreaks are primarily influenced by three factors, the pathogen, environment, and host, which form an epidemiological triangle influencing disease severity and pathogen dissemination (Scholthof, 2007). Co-evolution between these factors is a constant battle as new pathogens emerge, environmental conditions change, and hosts acquire immunity. Emerging pathogens arise as new serotypes or strains are created during pathogen replication or zoonotic transmission. The environment plays a critical role as changing migration patterns, sprawling urban development, and limited resources trigger natural and manmade crises. In the context of host susceptibility, vaccination is vital for boosting host immunity. However, challenges associated with manufacturing, distribution, and waning vaccine efficacy, contribute to host susceptibility.

The modern designation of a “black swan” event was proposed by Taleb (2007) and relies on three characteristics: (1) it is an outlier outside the scope of regular expectations; (2) it has an extreme impact; and (3) following the event, causes for the event can be rationalized. This concept can be applied to infectious disease outbreaks to describe rare but devastatingly large outbreaks. However, the criteria to define and forecast black swan disease outbreaks are still under discussion. Would black swan disease outbreaks arise due to a single factor or will they require convergence of multiple components of the epidemiological triangle? The ability to predict the *specific* outcome of disease outbreaks would help inform intervention strategies. Many efforts have been pursued to predict the occurrence and outcome of disease outbreaks; specifically, those with the potential to cause severe medical burden and negatively impacting society as a whole. However, additional investigation is necessary to better predict or even warn of *catastrophic large disease* outbreaks, which have the potential to be classified as a black swan event. Tools are needed that could at a minimum, provide timely warning, which would allow for accurate resource allocation and targeted control strategies.

Our previous research on historical outbreaks led to the development of Analytics for Investigation of Disease Outbreaks (AIDO; Velappan et al., 2019), a visual analytic tool which provides insights into the outbreak characteristics of 40 different infectious diseases. AIDO provides situational awareness during an unfolding outbreak of an infectious disease by comparing the user’s input data about the situation to a library of representative global outbreaks for that disease. AIDO provides the user with the closest matching historical outbreak that can help the user forecast the trajectory of their event. The analytic also provides detailed historic contextual data, capturing outbreak features and mitigation actions. For a single infectious disease, it is also possible to identify anomalous presentations of an unfolding outbreak by comparing the values of various properties (e.g., case counts) of the outbreak to the distribution of values for those properties of the representative library. This feature of AIDO was used in our current study to determine anomalous outbreaks with uncharacteristically high case counts. The tool also synthesizes data in our library to identify pathogen, environmental, and host features across diseases. We leveraged the large amount of data and analytics present in AIDO for 32 different infectious diseases to test our hypothesis that there may be common features from the pathogen, environment, or host epidemiological triad that characterize a potential infectious disease black swan event. We investigated whether there were (1) anomalous outbreaks that could be described for the diseases we researched, and (2) key *common* differentiating features that could be classified as warning signs of a potential black swan event. Here, we describe our study approach using AIDO, provide observations that support our hypothesis, and offer suggestions on how they could be used to develop new tools that provide alerts/warnings of potential black swan events in infectious disease. This study also illustrates the value of broad data analysis across the infectious disease realm to provide insights that may not be possible when we focus on singular infectious agents or diseases. We encourage the scientific community to develop new tools taking advantage of the vast and rich historical data to avoid repeating history.

## AIDO Application

### Identifying Potential Black Swan Outbreaks

**Figure 1:**
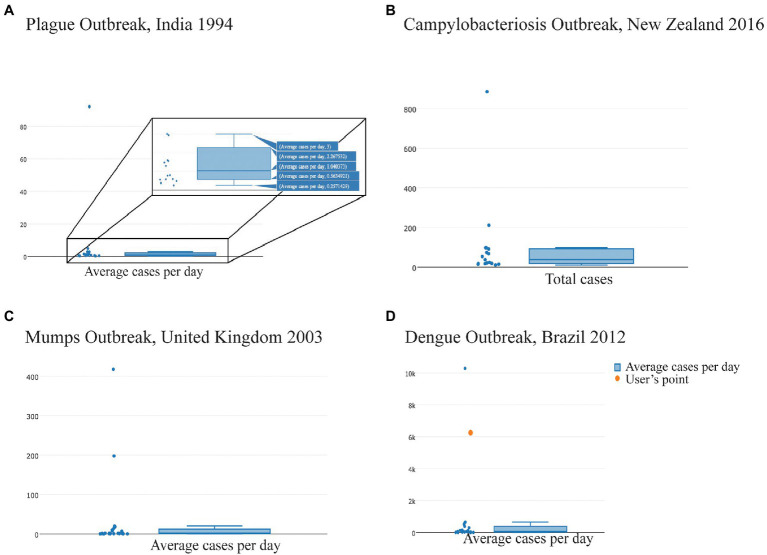
Analytics for Investigation of Disease Outbreaks (AIDO) generated property-specific box and whisker plots for each disease to visualize outliers from each disease-specific library of outbreaks. Titles for each panel identify the largest outlier outbreaks in the distribution; **(A)** Plague outbreak, India 1994—The hover feature shown provides the specific values (average, upper and lower quartile, and the upper and lower extremes) for average cases/day; **(B)** Campylobacteriosis outbreak, New Zealand 2016—Distribution of total case counts; **(C)** Mumps outbreak, United Kingdom 2003—Distribution of average cases/day; and **(D)** Dengue outbreak, Brazil 2012—an illustration of a user’s outbreak (shown as the orange dot) in the context of the library distribution for average cases per day.

Analytics for Investigation of Disease Outbreaks is available at https://aido.bsvgateway.org/ and contains ~650 outbreaks identified for about 40 diseases. Velappan et al. (2019) extensively describe the development of AIDO. One key analytic in this tool is anomaly detection, which provides a distribution of outbreak characteristics for a given disease across the representative outbreak library. We used the case count property to identify unusually large outbreaks for each infectious disease included in AIDO. The box and whisker plots show the total and average case counts for outbreaks of a given disease, including the upper and lower quartile values, the upper and lower extremes, and outliers to this distribution (Figure 1A). Figures 1B,C provide examples for the total case count distribution for campylobacteriosis and the average case count per day distribution for mumps, respectively. AIDO also allows visualization of the ongoing/unfolding outbreak in the anomaly detection algorithm by highlighting the “user’s input” as illustrated with dengue outbreaks in Figure 1D.

Using the AIDO disease libraries and anomaly detection algorithm, we identified potential black swan outbreaks (PBSO) for 32 infectious diseases. While AIDO has over 40 diseases in the database representing human, animal, and plant diseases, 32 have a sizeable representative library (more than 10 outbreaks) that could be used for analysis. We also focused on human diseases. We used the boxplots generated by AIDO in its anomaly detection feature, together with Tukey’s rule (Q3 + 1.5 × IQR), to identify outlier outbreaks (Tukey, 1977). The total case count for the largest outlier was compared to the average total case count values for that disease. Similar analyses were also performed with average case count/day (total case count/outbreak duration). The largest outliers showing at least an order of magnitude greater (>×10) for *both* characteristics, were marked as anomalous events. This criterion helped conservatively analyze the limited data sets we were working with.

In the next step, we compared the anomalous outbreaks to all other outbreaks in AIDO’s representative library for each disease. We used the outbreak descriptions provided in AIDO (event features, contextual information, and action or control measures) as well as the original data source for the given outbreak. Our goal here was to identify unique contributing and differentiating factors for the anomalous event and classify them as PBSOs based on the characteristics described by Taleb (2007).

### Cross-Disease Analysis

Our analysis identified PBSOs in 17 of the 32 diseases investigated, indicating that the potential for such events exists in diverse diseases caused by multiple categories of pathogens (bacteria, viruses, and parasites), diverse modes of transmission, and regardless of endemicity of a disease. Other than the PBSOs for Zika and the Novel influenza A, all other PBSOs occurred for diseases that were not newly emerging. Fifteen diseases did not have PBSOs as identified by our criteria: Brucellosis, Chikungunya, Crimean Congo Hemorrhagic fever, Japanese encephalitis virus, Monkey pox, Nipah virus, Norovirus, Polio, Q-fever, Rift Valley virus, Severe Acute Respiratory syndrome (SARS), Shigellosis, Salmonellosis, Tularemia, and West Nile Virus.

Diseases with PBSOs are shown in Table 1 together with information indicating how they met our criteria for PBSO classification (average total case count and average cases per day for the largest outlier outbreak compared to disease specific library). Differentiating factors for the PBSOs are captured under pathogen, environment, or host categories. Environment was further sub-categorized as natural (e.g., floods and outbreaks among wild animals) or manmade (e.g., poor healthcare infrastructure and war). We invite the reader to explore the specific unusual circumstances of these PBSOs on AIDO.^1^ Three diseases (malaria, STEC, and Zika) had PBSOs caused by convergence of pathogen, environmental, and host differentiating factors. Vaccine-preventable diseases, such as ebola, measles, mumps, novel influenza A, pertussis, rubella, yellow fever, and dengue (has vaccine development potential) had PBSOs caused by convergence of two differentiating factors from the epidemiological triad. These were either a convergence of host and pathogen, or host and manmade environmental factors (health infrastructure and behavioral factors such as vaccine hesitancy). Natural and/or manmade environmental factors contributed to outlier outbreaks in bacterial diseases: anthrax, campylobacteriosis, cholera, leptospirosis, meningococcal disease, and plague.

**Table 1:** Potential Black Swan Outbreaks (PBSOs) identified using Analytics for Investigation of Disease Outbreaks (AIDO).

		Total case count		Average cases/day		
Disease	PBSO					Differentiating factor(s) for PBSO
		PBSO	AIDO library (mean)	PBSO	AIDO library (mean)	
Anthrax	Zambia 2011	477	44	14	0.8	1. Environment—natural (wild animal outbreak) 2. Environment—manmade (behavioral—consumption of large quantities of contaminated meat)
Campylobacteriosis	New Zealand 2016	886	38	33	3	1. Environment—natural (flood) 2. Environment—manmade (physical infrastructure)
Cholera	Yemen 2017	1,854,483	5,903	2,324	32	1. Environment—manmade (behavioral, war and physical infrastructure, and geopolitical causes of lack of response)
Dengue	Brazil 2012	1,586,846	11,057	3,193	66	1. Pathogen—new serotype, higher vector density 2. Environment—manmade (public health infrastructure and decentralized surveillance)
Ebola	Sierra Leone 2014	8,893	119	19	1	1. Host—person-to-person transmission (high population density due to urban environment, unusual for ebola) 2. Environment—manmade (behavioral—lack of awareness and cultural practices)
Leptospirosis	Philippines 2012	7,687	156	23	2	1. Environment—natural (climate change) 2. Environment—manmade (behavioral—significantly large population displacement)
Malaria	Burundi 2000	536,000	92	1,178	1	1. Pathogen (drug resistance) 2. Host (malnutrition) 3. Environment—natural (heavy rains) 4. Environment—manmade (infrastructure)
Measles	DRC 2010	77,132	470	78	2.8	1. Host (insufficient vaccination) 2. Environment—manmade (physical infrastructure)
Meningococcal disease	Nigeria 2009	44,274	821	547	7	1. Environment—manmade (behavioral and infrastructure and geopolitical causes of inadequate response)
Mumps	UK 2003	70,203	207	418	1.7	1. Host—immunity gap (size of susceptible population—youth) 2. Environment—manmade (behavioral—vaccine hesitancy)
Novel Influenza A	USA 2009	112,079	51	469	0.4	1. Pathogen (new strain, H1N1) 2. Host (naïve population)
Pertussis	USA 2010	9,158	61	25	0.9	1. Host (vaccination age gap) 2. Environment—manmade (behavioral—lack of public awareness)
Plague	India 1994	460	19	92	1	1. Environment—manmade (behavioral—panic driven exodus)
Rubella	Poland 2012	22,520	241	134	2.5	1. Host (vaccine age gap) 2. Environment – manmade (behavioral, lack of public awareness)
STEC	Germany 2011	3,500	28	56	1	1. Pathogen (more virulent) 2. Host—person-to-person transmission (unusual) 3. Environment—manmade (behavioral, international commerce, food preparation method—uncooked food items, seeds, and sprouts)
Yellow fever	Nigeria 2017	4,242	59	19	0.4	1. Host (low vaccination) 2. Environment—manmade (public health infrastructure, urban transmission in addition to the normal sylvatic cycle)
Zika	Ceara, Brazil 2016	15,873	1,001	69	4.8	1. Pathogen (emerging virus, imported mosquito species) 2. Host (large naïve population) 3. Environment—manmade (public health infrastructure, behavior—early misdiagnosis)

While there were no pathogen or host factors common to *all* PBSOs, we noted that for almost every PBSO, the manmade environment (e.g., displacement, continuing cultural practices that promote transmission, and vaccine hesitancy) consistently contributed to the magnitude of the outbreak. The human behavior contributing to the lack of mitigation and control measures occurred independent of the quality of public health infrastructure or economic status of a location. PBSOs were identified globally and not restricted to specific areas of endemic disease.

In order for an outbreak to occur, it is necessary to have a convergence of a pathogen, a susceptible host, and an environment that promotes disease transmission. Our analysis of multiple diseases suggests that in order to identify warning signs for an impending PBSO, these same categories should be considered, albeit with a higher level of resolution to include qualifying questions for pathogen, environment, and host. It is also helpful to have an understanding of “normal” or baseline outbreak characteristics for a particular disease or syndrome, which could facilitate analysis of an unfolding event, as provided by the outbreak libraries of AIDO.

As an example, let us consider the very early stages of the Corona Virus Disease 2019 (COVID-19) pandemic, when a pneumonia surveillance program in China identified a novel pathogen which had not yet spread to the rest of the world. It was determined to be from the Coronavirus family by the time the world took notice, and therefore previous SARS and MERS outbreaks could be used to define some common characteristics. Using our AIDO libraries, we compared the largest outbreaks of SARS (Hong Kong and Guangdong, China, 2003) and MERS (South Korea, 2015) to the ongoing outbreak in China (WHO, 2020 Wind et al., 2020). The unfolding COVID-19 outbreak had almost 10 times more cases than the SARS outbreak in the timeframe being compared across the three outbreaks. Additionally, the case fatality rate was an order of magnitude lower. This indicated a more transmissible pathogen.

The following types of questions could be used to identify warning signs for an impending PBSO;

1. Pathogen – Is there a new variant/serotype detected? Is there drug resistance? Is there a new vector?
  Yes, a new type of Coronavirus, similar to SARS and MERS, but more transmissible. This indicated the potential for large outbreak.
2. Host – Is there is a significant susceptible population either due to vaccination gaps or poor public health infrastructure? Is there an unusual form of disease transmission?
  Yes, humans have never been exposed to this pathogen and had no pre-existing immunity, and the infectivity rate was higher compared to SARS and MERS. This again suggested there was potential for large outbreak.
3. Environment – Are there behavioral conditions that could promote large scale transmission (population density changes due to mass exodus, lack of awareness, and geopolitical barriers to response)
  Yes, the lack of awareness regarding the rate and mode of transmission occurred even as significant international travel continued. This too signified the potential for large outbreak.
  A “yes” to any of these questions could have provided warning signs at a very early stage prior to global spread.

In the case of COVID-19, all three factors had converged to cause the global pandemic. However, our analyses have shown that any one specific circumstance could provide a warning sign, as seen from the PBSO analysis across multiple diseases. There is no requirement for a convergence of the epidemiological triad as is necessary for an outbreak to occur.

## Discussion

Having PBSOs identified in 17 of the 32 diseases investigated in our study strongly suggests that all types of infectious outbreaks have the potential to become black swan events. One can use the epidemiological triad of pathogen, host, and the manmade environment to identify warning signs in the early stages of any infectious disease outbreak as described above. Our observations demonstrate that historical outbreak data can be a valuable source of information beyond just case counts and durations to inform epidemiological investigations. While our study was limited to data available in AIDO, it illustrates the value of mining rich repositories of outbreak information and the potential of computational tools that could be developed to automatically generate red flags for PBSOs. Such a tool could certainly be an important weapon in the arsenal for combating a new outbreak and preventing a PBSO by *targeted mitigation* as opposed to expensive, blanket actions.

There are limitations to our study. We took advantage of a tool (AIDO) and data set that was easily available to us. This is a small, but representative, data set that does not include every single outbreak that has occurred in the recent 20–30 years, and therefore, our observations could be biased. Some emerging diseases were not considered. Other diseases did not have PBSOs identified because there were limited libraries of outbreaks. Diseases like norovirus, which are self-limiting, did not pass our conservative criteria, because they did not have outbreaks with dramatically high case counts.

Despite the limited data set, we were able to identify warning signs that are applicable across the infectious disease realm, as evidenced by features of the manmade environment that contributed to each identified PBSO. Our analysis was possible due to AIDO’s anomaly detection feature, and the structured manner in which contextual data for outbreaks are captured.

Since the emergence of Severe acute respiratory syndrome–related coronavirus 2 (SARS-CoV-2), experts have contemplated designating the COVID-19 pandemic as a black swan event. Many, including Taleb (2007), point to the predicted pandemic potential of an emergent respiratory pathogen as evidence that COVID-19 was not a black swan event (Norman et al., 2020). Others argue that the indirect social and economic impacts during the pandemic are the true black swan events (Wind et al., 2020). However, it is important to note the warning signs that were identified in this ongoing discussion are very similar to the pathogen, environment, and host categories that we have highlighted in our study – notably the manmade environment (human behavior).

Through this perspective, we introduce a new area for risk modeling and call for future development of data analytics that could warn health officials of an impending PBSO. Our identification of the warning signs at the early stages of the COVID-19 pandemic suggests that a computational approach could be used to find nuanced indicators are that not immediately evident. By providing a risk measure that takes into account specific pathogen, manmade environment, and host features of an unfolding outbreak, such analytics can be companion tools to traditional, accurate epidemiological models and would facilitate effective, targeted mitigation actions, and specific resource allocation.

## Data Availability Statement

Underlying data on outbreaks used in this perspective are available at https://aido.bsvgateway.org/. Data can be accessed using the API provided on the landing page of the AIDO tool.

## Author Contributions

NV: methodology, investigation, data curation, writing – original draft, and visualization. KD-A: conceptualization, investigation, and writing – original draft. AD: supervision, conceptualization, and writing – original draft. All authors contributed to the article and approved the submitted version.

## Funding

This study was funded by USDA-ARS agreement ID NFE00060 subcontract to 58-0206-0-179.

## Conflict of Interest

The authors declare that the research was conducted in the absence of any commercial or financial relationships that could be construed as a potential conflict of interest.

## Publisher’s Note

All claims expressed in this article are solely those of the authors and do not necessarily represent those of their affiliated organizations, or those of the publisher, the editors and the reviewers. Any product that may be evaluated in this article, or claim that may be made by its manufacturer, is not guaranteed or endorsed by the publisher.

## Footnotes

^1^ https://aido.bsvgateway.org/

## Nomenclature

AIDO: Analytics for investigation of disease outbreaks
COVID-19: Corona virus disease 2019
SARS-CoV-2: Severe acute respiratory syndrome-related coronavirus 2
PBSO: Potential black swan outbreaks.
